# Tracking the Effect of Cathodal Transcranial Direct Current Stimulation on Cortical Excitability and Connectivity by Means of TMS-EEG

**DOI:** 10.3389/fnins.2018.00319

**Published:** 2018-05-15

**Authors:** Erica Varoli, Alberto Pisoni, Giulia C. Mattavelli, Alessandra Vergallito, Alessia Gallucci, Lilia D. Mauro, Mario Rosanova, Nadia Bolognini, Giuseppe Vallar, Leonor J. Romero Lauro

**Affiliations:** ^1^Ph.D. Program in Neuroscience, Department of Medicine and Surgery, University Milano-Bicocca, Monza, Italy; ^2^NeuroMi - Milan Center of Neuroscience, Milan, Italy; ^3^Department of Psychology, University of Milano-Bicocca, Milan, Italy; ^4^Department of Biomedical and Clinical Sciences “L. Sacco”, University of Milan, Milan, Italy; ^5^Fondazione Europea per la Ricerca Biomedica Onlus, Milan, Italy; ^6^Laboratory of Neuropsychology, IRCCS Istituto Auxologico, Milan, Italy

**Keywords:** cathodal tDCS, TMS-EEG, cortical excitability, posterior parietal cortex, neuromodulation

## Abstract

Transcranial direct current stimulation (tDCS) is increasingly used in both research and therapeutic settings, but its precise mechanisms remain largely unknown. At a neuronal level, tDCS modulates cortical excitability by shifting the resting membrane potential in a polarity-dependent way: anodal stimulation increases the spontaneous firing rate, while cathodal decreases it. However, the neurophysiological underpinnings of anodal/cathodal tDCS seem to be different, as well as their behavioral effect, in particular when high order areas are involved, compared to when motor or sensory brain areas are targeted. Previously, we investigated the effect of anodal tDCS on cortical excitability, by means of a combination of Transcranial Magnetic Stimulation (TMS) and Electroencephalography (EEG). Results showed a diffuse rise of cortical excitability in a bilateral fronto-parietal network. In the present study, we tested, with the same paradigm, the effect of cathodal tDCS. Single pulse TMS was delivered over the left posterior parietal cortex (PPC), before, during, and after 10 min of cathodal or sham tDCS over the right PPC, while recording HD-EEG. Indexes of global and local cortical excitability were obtained both at sensors and cortical sources level. At sensors, global and local mean field power (GMFP and LMFP) were computed for three temporal windows (0–50, 50–100, and 100–150 ms), on all channels (GMFP), and in four different clusters of electrodes (LMFP, left and right, in frontal and parietal regions). After source reconstruction, Significant Current Density was computed at the global level, and for four Broadmann's areas (left/right BA 6 and 7). Both sensors and cortical sources results converge in showing no differences during and after cathodal tDCS compared to pre-stimulation sessions, both at global and local level. The same holds for sham tDCS. These data highlight an asymmetric impact of anodal and cathodal stimulation on cortical excitability, with a diffuse effect of anodal and no effect of cathodal tDCS over the parietal cortex. These results are consistent with the current literature: while anodal-excitatory and cathodal-inhibitory effects are well-established in the sensory and motor domains, both at physiological and behavioral levels, results for cathodal stimulation are more controversial for modulation of exitability of higher order areas.

## Introduction

Although tDCS is one of the most used non-invasive brain stimulation techniques, there are relatively few studies addressing the mechanisms underlying its action (for a review see Stagg and Nitsche, 2011; Medeiros et al., 2012). A deeper understanding of the neurophysiological underpinnings of tDCS effects would be crucial to achieving a better refinement of stimulation protocols for clinical and research purposes.

Many hints on the neuronal mechanism of online and offline tDCS effects come from the translational approach of animal models. The first pioneering *in vitro* studies (Bindman et al., 1962, 1964; Creutzfeldt et al., 1962; Purpura and McMurtry, 1965) provided evidence for a polarity-dependent modulatory action, according to which anodal tDCS increase neurons' spontaneous firing rate and evoked potentials, whereas cathodal tDCS leads to the opposite effect. Recent *in vitro* animal models unveiled the complexity of tDCS effects, showing that the modulation of neuronal excitability results from the interaction of several factors, including the specific cell morphology and type, the interaction between neuronal compartments, the effects on afferent fibers and glial cells (Bikson et al., 2004; Radman et al., 2009; Gellner et al., 2016). When the duration of the stimulation exceeds 5 min, tDCS can induce long-lasting after-effects, presumably deriving from changes in synaptic strength. These after-effects are dependent upon continuous protein synthesis during stimulation (Gartside, 1968), likely mediated by mechanisms such as Long-Term Potentiation and Depression (LTP and LTD; Hattori et al., 1990; Moriwaki, 1991; Liebetanz et al., 2002; Fritsch et al., 2010; Ranieri et al., 2012; Rohan et al., 2015). The synaptic activity induced by tDCS increases Ca^2+^ intracellular amount, affecting calcium and sodium membrane channels (Islam et al., 1995), and it is dependent on enhanced brain-derived neurotrophic factor (BDNF) secretion and TrkB-activation (Fritsch et al., 2010).

The main limitation of studies with animal models is related to the issue of translating these findings to human beings, considering the relevant differences in the parameter, settings.

For instance, current intensity safety boundaries are set within 0.4–0.8 A/m^2^ (Nitsche et al., 2008) for humans, while animal studies intensity ranges between 5 to over 50 A/m^2^, often leading to inflammation, microglia activation and neurodegeneration on both anesthetized and alert animals (Rohan et al., 2015; Gellner et al., 2016; Koo et al., 2016; Monai et al., 2016; Podda et al., 2016).

In humans, the mechanisms underlying tDCS effects have been mainly investigated by means of pharmacological interventions (for a review: Medeiros et al., 2012), computational models of current flow (e.g., Miranda et al., 2013; Lafon et al., 2016), and recording stimulation-effects on cortical excitability and connectivity by means of other techniques. Among these, Functional Magnetic Resonance Imaging (fMRI; e.g., Stagg and Nitsche, 2011; Zheng et al., 2011), Positron Emission Tomography (PET; e.g., Lang et al., 2005), EEG (e.g., Accornero et al., 2007) and coupling TMS with Electromyography (e.g., Nitsche and Paulus, 2000), or EEG (e.g., Pellicciari et al., 2013; Romero Lauro et al., 2014; Bolognini and Miniussi, 2016) are the most common.

Robust evidence of tDCS-induced polarity-dependent shifts of cortical excitability have been shown by applying tDCS over the primary motor (M1; Priori et al., 1998; Nitsche and Paulus, 2000, 2001; Nitsche et al., 2003; Kirimoto et al., 2011), somatosensory (Kirimoto et al., 2011), and visual (Antal et al., 2004; Accornero et al., 2007) cortices, and measuring the amplitude of evoked activity.

In the domain of motor cortex, concerning Motor Evoked Potentials (MEPs) modulation, a certain amount of evidence suggests that the after-effects of both anodal and cathodal tDCS share a mechanism involving glutamatergic synapses (Stagg and Nitsche, 2011), but solely anodal tDCS plastic effects depend upon the modulation of GABAergic interneurons (Nitsche et al., 2004; Stagg et al., 2009; Stagg and Nitsche, 2011). Anodal tDCS indeed elicites a reduction of short-intracortical inhibition (SICI) and an increase in I-wave mediated intracortical facilitation (ICF), both measures of GABAergic interneuronal activity (Nitsche et al., 2005). Accordingly, by using Magnetic Resonance Spectroscopy (MRS) Stagg et al. (2009) demonstrated a reduction of GABA concentration within M1 10 min after anodal tDCS.

Beyond modulating the excitability of the stimulated area, tDCS can also alter cortical connectivity from the targeted area, thus affecting broader brain networks. Stagg et al. (2013) showed that anodal tDCS increased perfusion in a wide set of brain areas including the left primary sensory cortex (S1), the midcingulate cortex, the paracingulate cortex and the left parietal cortex. It was also found an increased connectivity between the stimulated area and the contralateral homologous one (i.e., right DLPFC) and the left sensorimotor cortex, but a decreased connectivity with the bilateral thalamus. Cathodal tDCS, instead, decreased perfusion in the bilateral thalami and right middle and inferior temporal gyri and led to a decrease of connectivity between the left DLPFC and an extensive region in the left temporal, parietal, and occipital lobes. In contrast, an increased functional connectivity was observed after cathodal but not anodal tDCS over M1 within the motor and non-motor network, such as the default mode brain network (Amadi et al., 2014).

Recently, the effects on cortical excitability and connectivity of anodal tDCS applied on non-motor areas were tracked using TMS-EEG. Thorugh the activation of a cortical area with TMS, it is possible to record in real time the cortical response over the cortex by means of HD-EEG recordings. TMS-evoked potentials (TEPs) recorded with this technique reflect cortical excitability and connectivity of the targeted area, representing a direct measure of the neural state. TMS-EEG studies on tDCS effects on cortical excitability showed an increase of TEPs during and 10 min after the end of anodal tDCS, which were not restricted to the stimulated area, but rather affected different cortical networks according to the brain activation state. Effects spread following structural connections at resting state (Romero Lauro et al., 2014, 2015), whereas they were confined to functionally relevant areas when tDCS was applied during task execution (Pisoni et al., 2017).

In the present study, we aimed at further complementing previous knowledge on the neurophysiological basis of tDCS by applying the same TMS-EEG co-registration approach to explore the effects induced by cathodal tDCS. Addressing the cortical effects of cathodal tDCS acquires **a critical** relevance considering that the behavioral outcomes of this stimulation are more uncertain in comparison to those induced by anodal tDCS (e.g., Jacobson et al., 2012 for a review). Indeed, whereas polarity-dependent opposite effects – anodal excitatory and cathodal inhibitory–, are usually reported when stimulating the primary sensory, motor or visual cortices (but see, e.g., Batsikadze et al., 2013; Sczesny-Kaiser et al., 2016 for different results), the evidence becomes more controversial when higher cognitive functions and the underlying brain areas are targeted (Jacobson et al., 2012). Typically, anodal tDCS has been found to enhance the targeted cognitive function, whereas cathodal stimulation is reported as less effective or is not explored at all. For instance, recent evidence-based guidelines for clinical use of tDCS (Lefaucheur et al., 2017) do not include cathodal stimulation for any disease.

To mirror previous data (Romero Lauro et al., 2014, 2015), in the present study we replicate the methodology but reversing the stimulation polarity applying cathodal tDCS over the right PPC, and tracking its effects on cortical excitability and connectivity performing TMS-EEG co-registrations before, during and 10 min after the end of the stimulation. Since our participants did not take part in the previous study, we decided to not directly compare the three groups (anodal, cathodal, and sham), rather we compared stimulation effects within the same stimulation condition; i.e., pre-post cathodal tDCS and pre-post sham stimulation. We hypothesized a reduction of cortical excitability and connectivity, during and following the stimulation.

## Materials and methods

### Participants

Fifteen healthy, right-handed volunteers (five males, mean age 25.4 years, *SD* 3.5, range 21–32) participated in the study. To define the sample size, we run a-priori computations in GPower to determine the number of participants needed to highlight a potential effect of stimulation with a power of 0.90 and an alpha level of *p* = 0.05. In detail, we ran two a-priori sample size computations, one with the smallest significant effect found in Romero Lauro et al. (2014), and the other with the mean value of the reported significant effect sizes (eta squared of 0.34 and 0.42, respectively). Each participant completed an Adult Safety Screening Questionnaire (Keel et al., 2001), and gave informed written consent before the experiment. Participants did not report contraindications to non-invasive brain stimulation (Rossi et al., 2009), namely no history of medical disorders, no substance abuse, no use of central nervous system-effective medication, no psychiatric and neurological disorders, including brain surgery, tumor, or intracranial metal implantation. The study took place in the TMS-EEG laboratory of the University of Milano-Bicocca, was approved by the local Ethics Committee, and it was carried out in accordance with the ethical standards of the revised Helsinki Declaration.

### Procedure

For each participant, the experimental session consisted of three blocks of TMS-EEG recordings performed before (pre-tDCS), during (during-tDCS) and 10 min after cathodal tDCS (post-tDCS) applied over the right PPC. Each recording lasted about 7 min during which participants were in a resting condition, fixating a white cross in a black screen (17′′). The second group of 15 participants took part in a control Sham session. Six participants of this sample were taken from a previous study (Romero Lauro et al., 2014), while the remaining nine were recruited from the present sample. Sham sessions were identical to the cathodal ones, but tDCS was turned off 30 s after the start. The order of the two sessions (cathodal and sham tDCS) was counterbalanced across subjects.

### TMS stimulation

TMS was delivered with an Eximia™ TMS stimulator (Nexstim™, Helsinki, Finland) using a focal figure-of-eight bi-pulse 70 mm-coil. As in Romero Lauro et al. (2014), stimulation target was the left PPC, between P1 and CP1 EEG electrodes. High-resolution (1 × 1 × 1 mm) structural magnetic resonance images (MRI) were acquired for each participant using a 3 T Intera Philips body scanner (Philips Medical Systems, Best, NL). TMS target was identified on individual MRIs using a Navigated Brain Stimulation (NBS) system (Nexstim™, Helsinki, Finland), which employs infrared-based frameless stereotaxy to map the position of the coil and participant's head, within the reference space of the individual's MRI space. Mean MNI coordinates for the target site were X = −31 (*SD* = 5.2) Y = −70 (*SD* = 6.6) Z = 54 (*SD* = 3.7). The NBS system allowed to continuously monitoring the position and orientation of the coil, thus assuring precision and reproducibility of the stimulation across sessions. Moreover, the NBS system estimated on-line the distribution and intensity (V/m) of the intracranial electric field induced by TMS. It uses a locally best-fitting spherical model, accounting for the head and brain shape of each participant, and taking into consideration the distance from scalp, coil position, and orientation. Mean stimulation intensity, expressed as a percentage of the maximal output of the stimulator, was 58% (range = 50–63%), corresponding to an electric field of 100 ± 14 V/m. The coil was placed tangentially to the scalp, and adjusted for each participant in order to direct the electric field perpendicularly to the shape of the cortical gyrus, following the same procedure of previous studies (Casarotto et al., 2010; Mattavelli et al., 2013; Romero Lauro et al., 2014). Since TMS over parietal sites can activate temporal and frontal muscles, hence eliciting artifacts in the EEG recordings, the site of the stimulation was individually adjusted, in order to avoid or reduce as much as possible muscle twitches. TMS pulses were delivered at an inter-stimulus interval (ISI) randomly jittered between 2,000 and 2,300 ms (0.4–0.5 Hz). One hundred and eighty TMS pulses were delivered for each block.

### tDCS parameters

**Figure 1:**
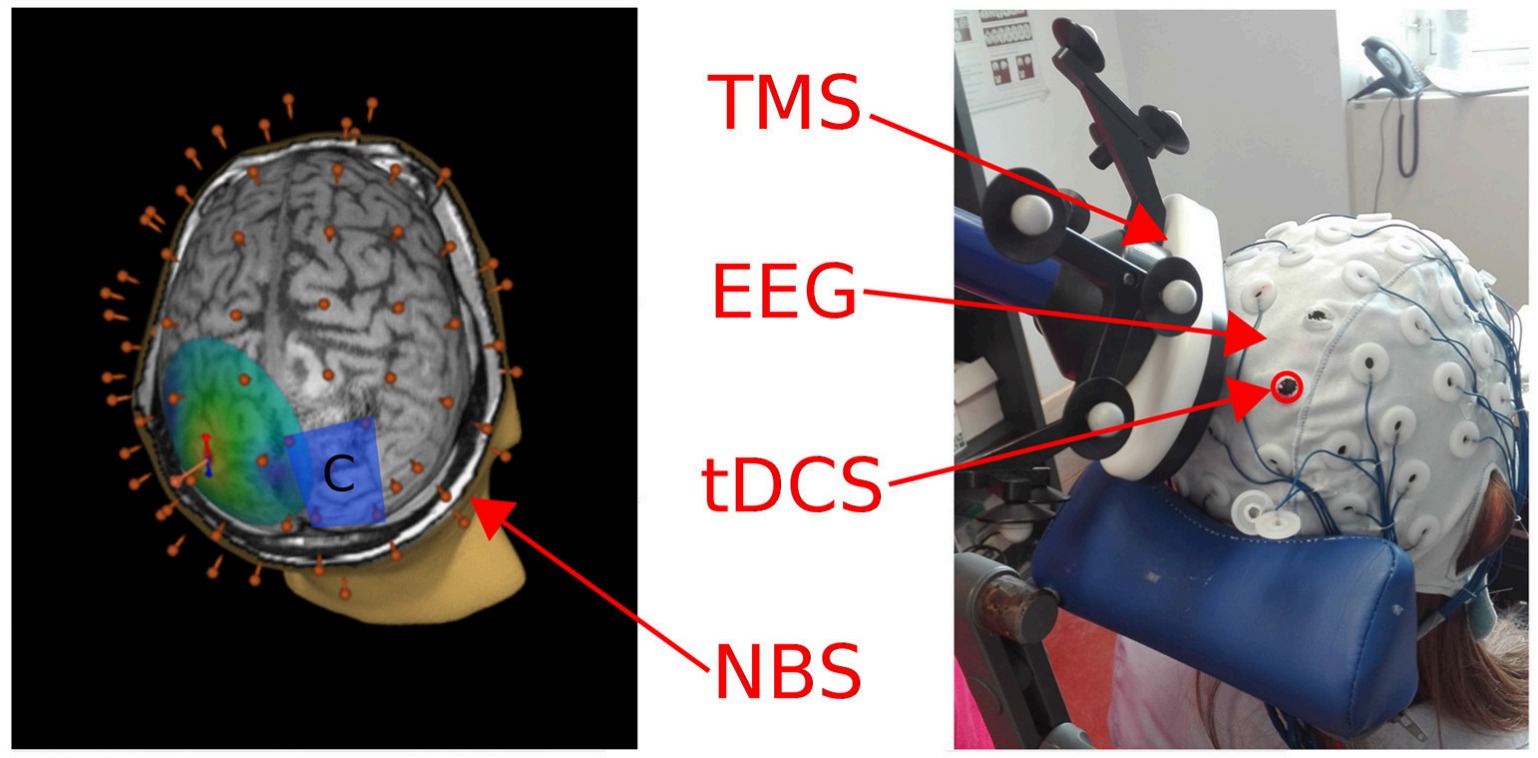
**Left**: A 3D reconstruction of the individual MRI is depicted, showing the electrical field induced by TMS on the left parietal cortex. The blue rectangle represents the cathode patch position over the right parietal cortex, the 60 red points correspond to the position of the EEG cap electrodes. **Right**: A picture of the experimental setting used to deliver tDCS and TMS concurrently while recording EEG (during-tDCS condition).

tDCS was delivered by a battery-driven constant current stimulator (Eldith™, Neuroconn, Ilmenau, Germany) using a pair of rubber electrodes and a conductive paste (Ten20 conductive EEG paste, Kappamedical™, USA) to attach them to participants' head and reduce impedance. An intracephalic montage was used. The cathode (size = 9 cm^2^; current density = 0.08 mA/cm^2^) was placed over the right PPC under the EEG cap, in a site corresponding to P2 electrode, which was previously removed from the cap together with the CP2 electrode, as in Romero Lauro et al. (2014). The anode (size = 25 cm^2^, current density = 0.03 mA/cm^2^) was positioned over the left supraorbital area. A constant current of 0.75 mA was applied for 15 min, with 8 s of fade-in/fade-out period. Different sized electrodes were used to increase the focality of stimulation (Nitsche et al., 2008). For sham tDCS, the same electrodes arrangement and stimulation parameters were used, but the stimulator was turned off after 30 s (Gandiga et al., 2006). The feasibility of concomitant EEG recording and tDCS application has been recently probed (Wirth et al., 2011; Faria et al., 2012; Schestatsky et al., 2013). In order to avoid tDCS induced artifacts in the EEG trace, the tDCS electrodes and the conductive gel never came in contact with the surrounding EEG recording leads and they were far away from the ground electrodes (see Figure 1). Transient EEG artifacts were observed only during the fade-in and fade-out phases of tDCS stimulation, while TMS-EEG trials were never affected by those transient artifacts. The study was performed in single blind, and no adverse effects were reported.

### EEG recording data during TMS

TEPs were continuously recorded using a TMS compatible 60-channels amplifier (Nexstim Ltd., Helsinki, Finland), which gates the TMS artifact and prevents saturation by means of a proprietary sample-and-hold circuit (Virtanen et al., 1999). EEG signals were referenced to two electrodes placed over the forehead and used as ground. Eye movements were recorded with two additional electrodes placed near the eyes, in order to monitor ocular artifacts both in the vertical and horizontal axes. As in previous studies (Massimini et al., 2005; Casarotto et al., 2010), in order to prevent auditory potentials due to TMS pulses, a masking noise, which reproduced scrambled TMS “click” time varying frequency components, was continuously played into earplugs worn by participants during the experimental sessions. Electrodes impedance was kept below 5 kΩ, and EEG signals were recorded with a sampling rate of 1,450 Hz.

### EEG data analysis

EEG data were pre-processed using Matlab R2016b® (Mathworks, Natick, MA, USA). First, recordings were down-sampled to 725 Hz. Continuous signal was then split in single trials, from 800 ms before to 800 ms after the TMS pulse. Trials with artifacts due to eye blinks/movements, or spontaneous muscle activity were removed following a semi-automatic procedure (Casali et al., 2010), and the visual inspection of the signal by trained experimenters (AP, EV). The average number of trials considered in the analysis was 123 (*SD* ± 3) for the pre-tDCS, 123 (*SD* ± 5) for the during tDCS block, and 122 (*SD* ± 3) for the post-tDCS condition. TEPs were computed by averaging selected artifact-free single trials and by filtering them between 2 and 40 Hz. Bad or missing channels, as P2 and CP2 for each session, were interpolated using the spherical interpolation function of EEGLAB (Delorme and Makeig, 2004). TEPs were then average referenced and baseline corrected between −300 and −50 ms before the TMS pulse. For each condition, as an index of global excitability, Global Mean Field Power (GMFP, Casarotto et al., 2010; Romero Lauro et al., 2014, 2015; Pisoni et al., 2017) was computed on averaged TEPs of 60 channels for three temporal windows defined in an interval between 0 and 150 ms from TMS pulse onset. The three time windows were: 0–50, 50–100, and 100–150 ms. To further identify the specific contributions of different cortical regions to the modulation of global cortical excitability, indexes of local excitability (Local Mean Field Power, LMFP) were measured following the same procedure used for GMFP. Four clusters of electrodes, with 4 electrodes each, were selected based on anatomical locations. Two parietal clusters: the left one corresponding to TMS hotspot (CP1, CP3, P1, and P3), and the right one corresponding to the area covered by the tDCS cathode (CP2, CP4, P2, and P4). Two frontal clusters corresponded to the areas structurally and functionally connected to the parietal ones: the left frontal cluster (F1, F5, FC1, and FC3) and the right frontal cluster (F2, F6, FC2, and FC6). In order to obtain a synthetic index of global and local cortical excitability, GMFP and LMFP values were cumulated within the three time windows (0–50, 50–100, and 100–150 ms after the TMS pulse) and for each experimental condition (pre-, during-, and post-tDCS).

### Source modeling

Source modeling was performed in order to assess the impact of tDCS on cortical excitability, avoiding the potential confound of volume conduction and allowing a better definition of the spatial distribution of the tDCS effects (as in Romero Lauro et al., 2015). The analysis was run on 14 out of the 15 participants enrolled in the experiment, since in one of the subjects MRI data were not suitable for source reconstruction (see for details on the procedure see Casali et al., 2013). First, meshes of cortex, skull, and scalp compartments (containing 3,004, 2,000, and 2,000 vertices, respectively) were obtained following the 3-spheres BERG method (Berg and Scherg, 1994), as implemented in the Brainstorm software package (http://neuroimage.usc.edu/brainstorm). This method includes 3 concentric spheres with different homogeneous conductivities, representing the best-fitting sphere of inner and outer skull and scalp. The model was constrained to the meshes of these tissues obtained from the individual MRIs of the experimental subjects in the Statistical Parametric Mapping software package (SPM5, http://www.fil.ion.ucl.ac.uk/spm/software/spm5/): for each participant, binary masks of skull and scalp obtained from individual MRIs were warped to the canonical meshes of the Montreal Neurological Institute (MNI) atlas. Then, the inverse transformation was applied to the MNI mesh of the cortex for approximating to real anatomy. The cortex, in particular, was reconstructed as a 3D grid of 3004 fixed dipoles, normally oriented with respect to the cortical surface. For each participant, EEG sensor position was aligned to the canonical anatomical markers (pre-auricular points and nasion), and the forward model was computed. The inverse solution was computed on the average of all artifact-free TMS-EEG trials using the weighted minimum norm estimate with smoothness prior, following the same procedures as in Casali et al. (2010). This method is advantageous because it provides stable solution also in the presence of noise (Silva et al., 2004), and it does not require any a priori assumption about the nature of the source distribution (Hämäläinen and Ilmoniemi, 1994). After source reconstruction, a statistical threshold was computed in order to assess when and where the post-TMS cortical response differed from pre-TMS activity (i.e., to identify TMS-evoked response). To do so, a non-parametric permutation-based procedure was applied (Pantazis et al., 2003). A binary spatial-temporal distribution of statistically significant sources was obtained and thus only information from significant cortical sources was used for further analyses. As a measure of global cortical activation, we cumulated the absolute Significant Current Density (global SCD, measured in mA/mm^2^, Casali et al., 2010) overall 3,004 cortical vertexes and over the three time windows (0–50, 50–100, and 100–150 ms) for each recording session (pre-tDCS, during-tDCS and post-tDCS). Finally, in order to mirror the LMFP EEG data analysis of the study, for each time window and each experimental condition, a local SCD was computed in the vertexes within four different Brodmann's areas (BAs), identified by means of an automatic tool of anatomical classification (WFUPickAtlas tool; http://www.ansir.wfubmc.edu). These BAs approximately corresponded to the original four clusters of LMFP (left/right BA 6 and 7).

### Statistical analyses

To estimate whether tDCS affected global or local cortical excitability, GMFP and LMFP values were submitted to a series of linear mixed effects models (Baayen et al., 2008) in R statistical computing software environment (R Core Team, 2014) with the “lme4” package (version 0.6-82, Bates et al., 2014). In particular, GMFP was considered as a continuous dependent variable, while *Condition* (factorial, 3 levels: pre-, during- and post-tDCS) and *Time Window* (factorial, 3 levels: 0–50; 50–100; and 100–150 ms) were tested as fixed factors. The by-subject intercept was included as random factor. The inclusion of a main effect or interaction in the final model was assessed by means of Likelihood Ratio Test (LRT, see Baayen et al., 2008), including a parameter if it significantly increased the model's goodness of fit. The same procedure was adopted for LMFP values, which were analyzed separately for each electrodes cluster. A further test with a Bayesian ANOVA on the same values was performed to test for the null hypothesis (Rouder et al., 2009; Etz et al., 2018) by means of the Bayesian ANOVA analysis using “JASP” software environment (version 0.8.2.0, JASP Team, 2017).

The same analysis was performed for source modeling data on global and local SCD values.

The whole procedure was also adopted to analyze sham session data.

## Results

### Cathodal stimulation—sensor analysis

#### GMFP

**Figure 2:**
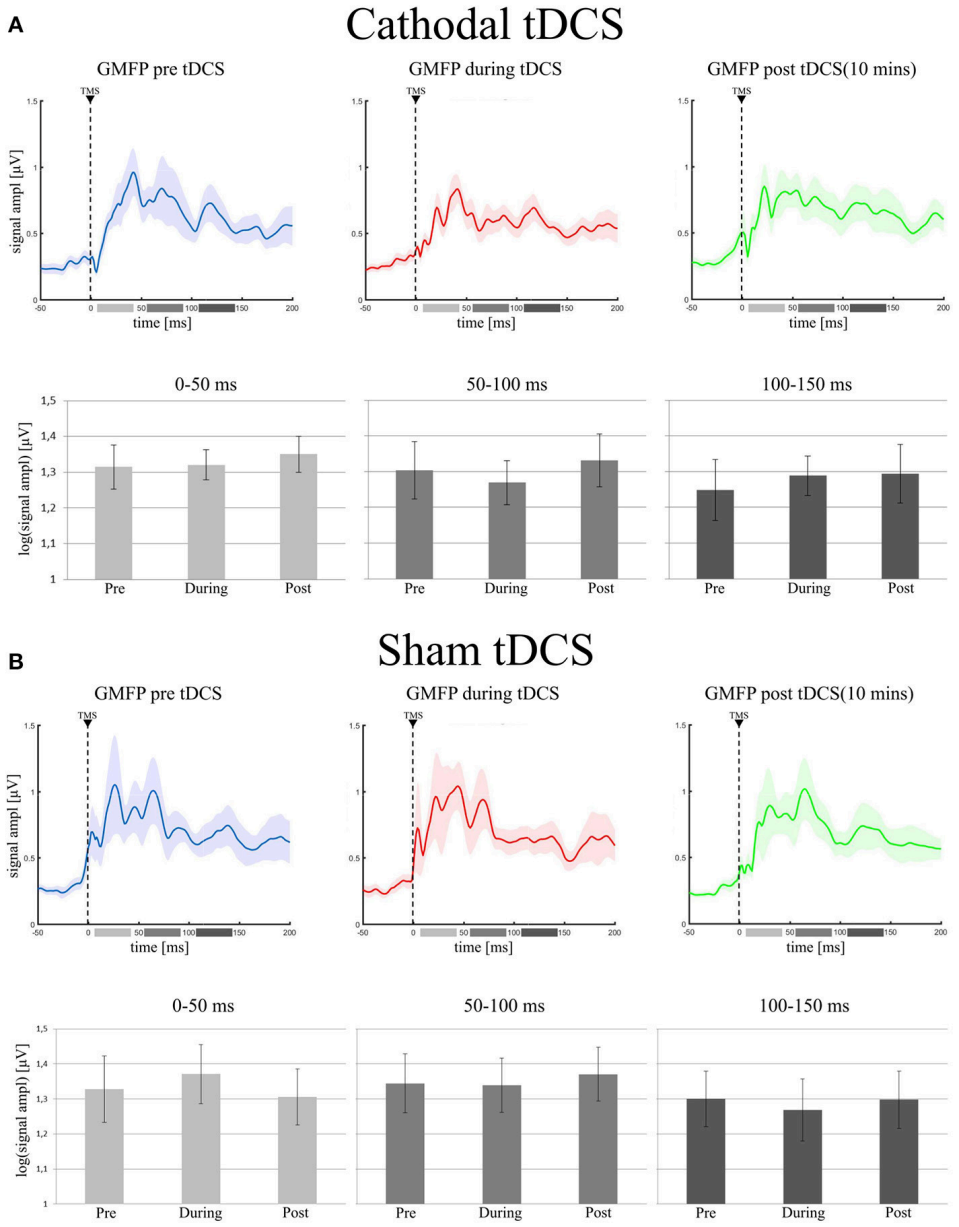
**(A)** (Cathodal tDCS): in the upper row it is shown the Grand Average of GMFP in the three experimental conditions (pre tDCS = blue trace; during tDCS = red trace; post tDCS = green trace). Shadowed areas represent ± SE. In the lower row, the bar histograms represent the mean values of the log-GMFP in the three time-windows of our interest (0–50 ms = light gray, 50–100 ms = gray, 100–150 ms = dark gray) for each recording session. The error bars represent ± SE. **(B)** (Sham tDCS) shows the same data, but for the sham group: the Grand Average of GMFP and the mean values of log-GMFP, in the upper and lower row, respectively.

The final model on GMFP values did not include the main effect of *Condition* in the 0–50 ms [$\chi_{\left(2\right)}^{2}$ = 1.15; *p* = 0.56], in the 50–100 ms [$\chi_{\left(2\right)}^{2}$ = 0.26; *p* = 0.87] and in the 100–150 ms [$\chi_{\left(2\right)}^{2}$ = 1.62; *p* = 0.44] time windows. GMFP thus did not change when recorded before, during or after cathodal tDCS (see Figure 2A). Bayesian analysis, indeed, provided moderate support in favor of the null hypothesis for the first (BF_01_ = 5.1), the second (BF_01_ = 4.6), and the third (BF_01_ = 5.3) time window.

#### LMFP

**Figure 3:**
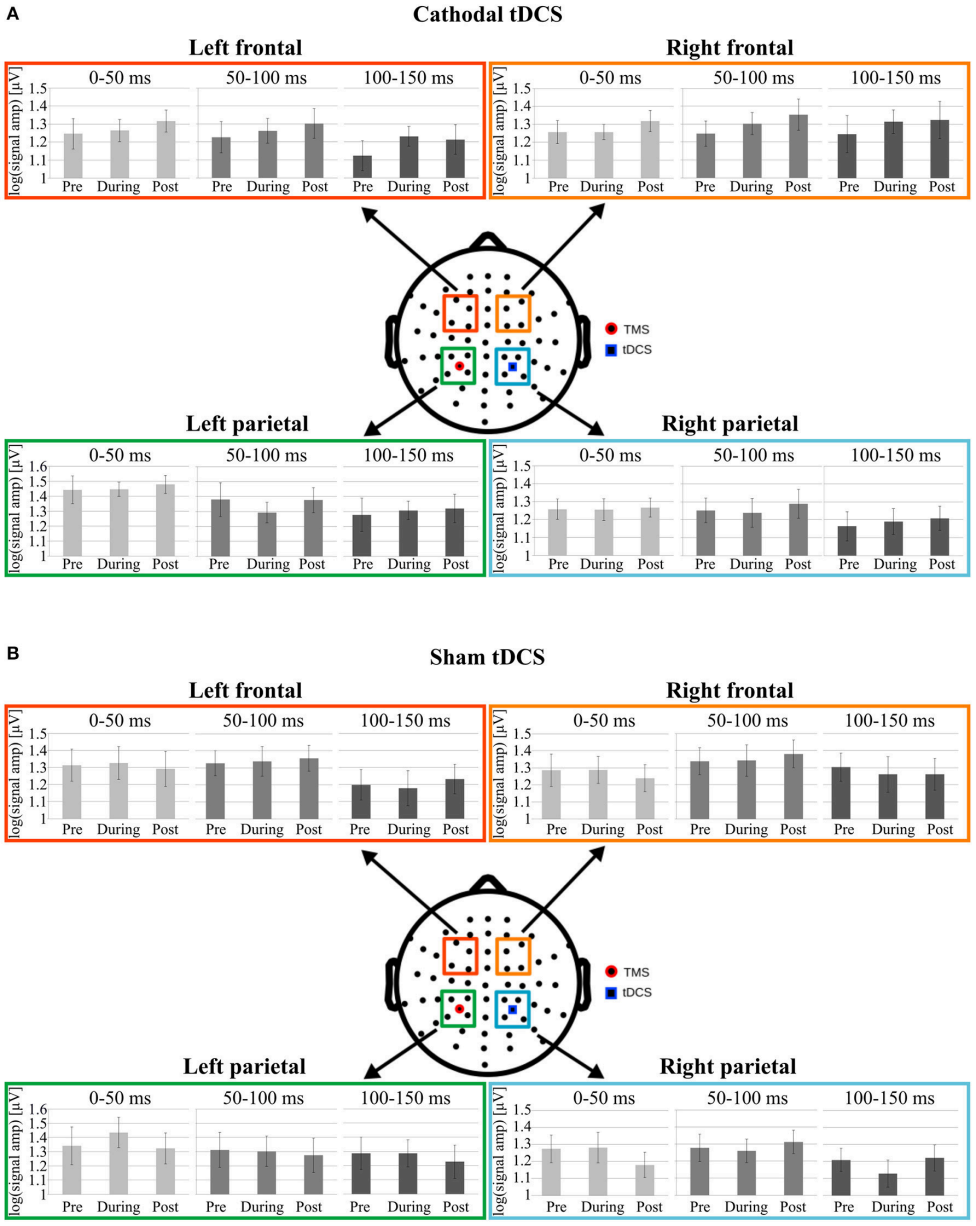
Data from Cathodal and Sham groups are respectively shown in **(A,B)**. Mean log-LMFP for the four clusters of interest. Colored squares on the head model represent the electrodes clusters in the parietal and frontal regions. The blue square in the right parietal region represents the location of the tDCS cathode, whereas the red dot in the left parietal cluster represents the TMS position. For each cluster, the bar graphs represent mean log-LMFP in the baseline, during and post tDCS conditions, for the three temporal windows: 0–50 ms (light gray), 50–100 ms (gray), and 100–150 ms (dark gray). Error bars represent ± SE.

Analyses run on LMFP values did not support any effect of cathodal tDCS in any of the considered clusters of electrodes (see Figure 3A).

In particular, concerning C1, the cluster under the tDCS cathode, LRT indicated not to include *Condition* in the model run on LMFP values in the first [$\chi_{\left(2\right)}^{2}$ = 1.53; *p* = 0.46], second [$\chi_{\left(2\right)}^{2}$ = 0.63; *p* = 0.73], and third [$\chi_{\left(2\right)}^{2}$ = 1.36; *p* = 0.51] time window. Critically, Bayesian analyses moderately supported the null hypothesis, indicating no effect of tDCS on LMFP computed in this cluster for the first (BF_01_ = 5.5), the second (BF_01_ = 3.9) and the third (BF_01_ = 5.7) time window.

Analyzing C2, the cluster under the TMS coil, LRT indicated to not include *Condition* in models for any time window [0–50 ms: $\chi_{\left(2\right)}^{2}$ = 1.12; *p* = 0.57; 50–100 ms: $\chi_{\left(2\right)}^{2}$ = 0.99; *p* = 0.61; 100–150 ms: $\chi_{\left(2\right)}^{2}$ = 1.22; *p* = 0.54]. Crucially, Bayesian analyses moderately supported the null hypothesis, indicating no effect of tDCS on LMFP computed in this cluster in any time window (0–50 ms: BF_01_ = 6; 50–100 ms: BF_01_ = 5.3; 100–150 ms: BF_01_ = 5.5).

Similarly, in C3 LRT indicated to not include *Condition* in models for any time window [0–50 ms: $\chi_{\left(2\right)}^{2}$ = 1.03; *p* = 0.6; 50–100 ms: $\chi_{\left(2\right)}^{2}=$ 2.78; *p* = 0.25; 100–150 ms: $\chi_{\left(2\right)}^{2}$ = 1.08; *p* = 0.58]. Yet, Bayesian analyses moderately supported the null hypothesis, indicating no effect of tDCS on LMFP computed in this cluster in any time window (0–50 ms: BF_01_ = 3.8; 50–100 ms: BF_01_ = 3; 100–150 ms: BF_01_ = 4.9).

The final model on C4 values did not include the main effect of *Condition* in the 0–50 ms [$\chi_{\left(2\right)}^{2}$ = 1.4; *p* = 0.49] in the 50–100 ms [$\chi_{\left(2\right)}^{2}$ = 2.38; *p* = 0.30] or in the 100–150 ms [$\chi_{\left(2\right)}^{2}$ = 3; *p* = 0.22] time windows. Even in this case, Bayesian analysis supported moderately the null hypothesis, indicating no effect of tDCS on LMFP computed in this cluster in any time window (0–50 ms: BF_01_ = 4.5; 50–100 ms: BF_01_ = 4.4; 100–150 ms: BF_01_ = 3.5).

### Cathodal stimulation—source modeling analyses

Source modeling analyses confirmed results from the sensor analyses. A full report of statistical results of analyses run on Global and Local SCD is reported in Table 1.

**Table 1:** List of *p* values resulting from the source modeling analysis performed for each time window (0–50, 50–100, and 100–150 ms) within the four Brodmann's areas (BAs), that corresponded approximately to the four clusters.

	**BA 7 L**	**BA 7 R**	**BA 6 L**	**BA 6 R**
**CATHODAL**				
0–50 (ms)	*p =* 0.736	*p =* 0.729	*p =* 0.513	*p =* 0.605
50–100 (ms)	*p =* 0.232	*p =* 0.204	*p =* 0.683	*p =* 0.787
100–150 (ms)	*p =* 0.484	*p =* 0.582	*p =* 0.835	*p =* 0.656

*In particular, BA 7 L is the Left parietal cluster (TMS site; CP1, CP3, P1, and P3); BA 7 R is the Right parietal cluster (tDCS cathode; CP2, CP4, P2, and P4); BA 6 L is the Left frontal cluster (F1, F5, FC1, and FC3); BA 6 R is the Right frontal cluster (F2, F6, FC2, and FC6)*.

**Figure 4:**
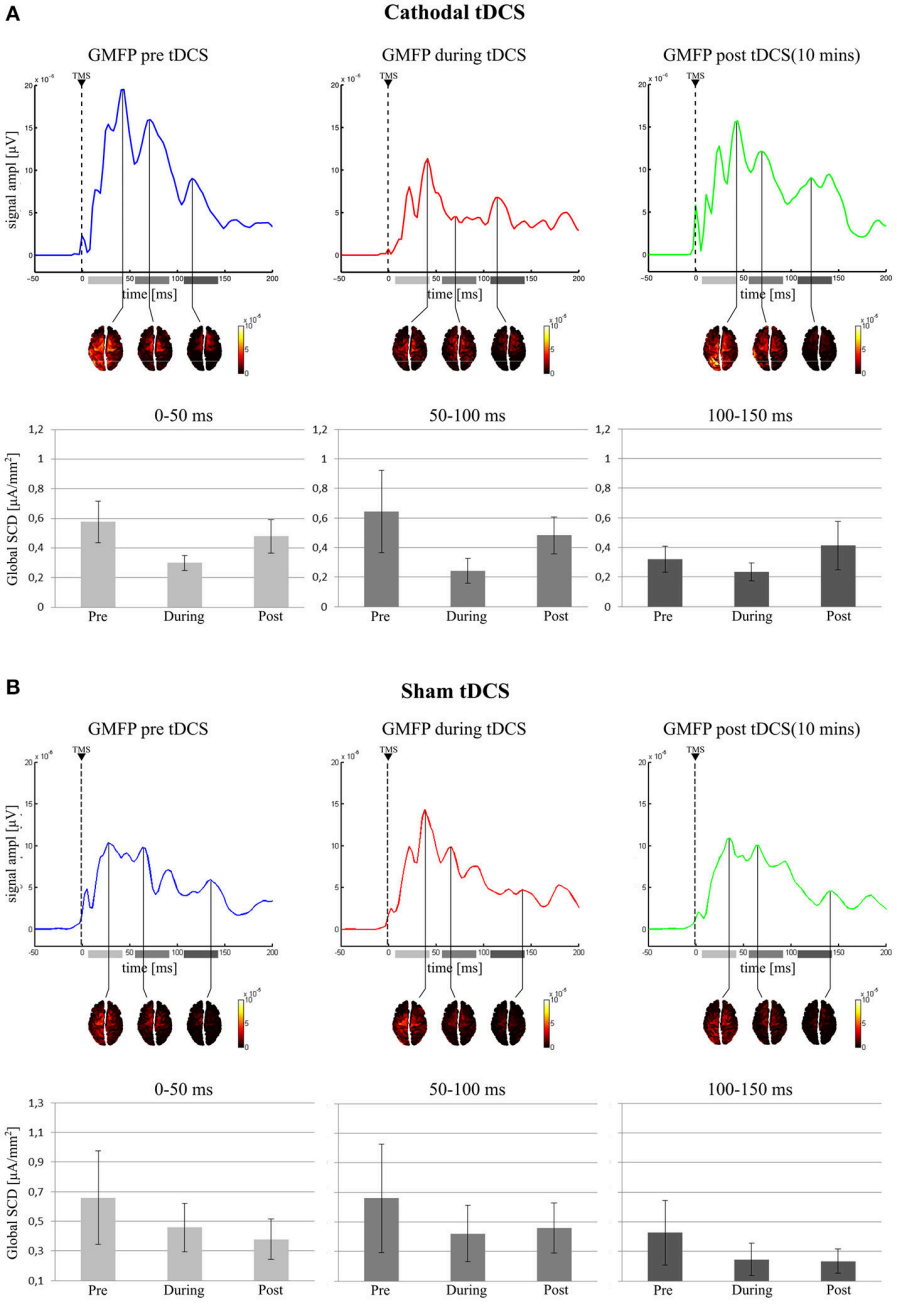
Active vertexes and current spread at the local maxima in the GMFP for the three time-windows. In the **(A)** (Cathodal tDCS), for each recording session, the GMFP is shown on first top row, with the area beneath the curve divided in the three tested time windows (0–50 ms: light gray; 50–100 ms: gray; 100–150 ms: dark gray). The second row shows the estimated cortical sources in time coincidence with the maximum GMFP value, for each time window. In the last row are reported the mean values of the Global SCD for each condition and in each time window. **(B)** (Sham tDCS) shows the same results but for the sham group.

The final model on Global SCD did not include *Condition* in any time window, indicating no effect of cathodal tDCS on cortical activation induced by TMS (see Figure 4A).

Similarly, for both left and right BA7 LRT indicated no inclusion of the factor *Condition* in the final model, suggesting no influence of cathodal tDCS on local cortical activity in the left and right parietal cortices.

The same result holds for the left and right BA6, where no effect of tDCS was highlighted on local SCD.

### Sham stimulation—sensor analysis

#### GMFP

As expected, sham stimulation did not modulate global indices of cortical excitability. In particular, the final model run on GMFP did not include the main effect of *Condition* in any time window [0–50 ms: $\chi_{\left(2\right)}^{2}$ = 1.2; *p* = 0.55; 50–100 ms: $\chi_{\left(2\right)}^{2}$ = 0.43; *p* = 0.81; 100–150 ms: $\chi_{\left(2\right)}^{2}$ = 0.18; *p* = 0.91 see Figure 2B]. Bayesian analyses, moderately supported the null hypothesis for the inclusion of the factor *Condition* in the final model (0–50 ms: BF_01_ = 3.9; 50–100 ms: BF_01_ = 5.1; 100–150 ms: BF_01_ = 5.4).

#### LMFP

Analyses run on LMFP confirm the lack of any effect of sham tDCS in any of the considered clusters of electrodes in modulating local cortical excitability (see Figure 3B).

In C1, LRT values were non-significant for the first [$\chi_{\left(2\right)}^{2}$ = 2.4; *p* = 0.3], the second [$\chi_{\left(2\right)}^{2}$ = 0.98; *p* = 0.61] and the third [$\chi_{\left(2\right)}^{2}$ = 2.29; *p* = 0.32] time window. Bayesian analyses moderately supported the null hypothesis, indicating no effect of sham tDCS on LMFP computed in this cluster (0–50 ms: BF_01_ = 2.3; 50–100 ms: BF_01_ = 4.73; 100–150 ms: BF_01_ = 3.3).

The same holds for C2. LRT values were not significant for the factor *Condition* in any time window [0–50 ms: $\chi_{\left(2\right)}^{2}$ = 1.28; *p* = 0.53; 50–100 ms: $\chi_{\left(2\right)}^{2}$ = 0.86; *p* = 0.65; 100–150 ms: $\chi_{\left(2\right)}^{2}$ = 0.2; *p* = 0.9]. Similarly, Bayesian analyses moderately supported the data under the null hypothesis for the first (BF_01_ = 3.4), the second (BF_01_ = 5.4), and the third (BF_01_ = 5) time window.

Similarly, in C3 LRT indicated not to include factor *Condition* for any time window [0–50 ms: $\chi_{\left(2\right)}^{2}$ = 1.94; *p* = 0.38; 50–100 ms: $\chi_{\left(2\right)}^{2}$ = 0.74; *p* = 0.69; 100–150 ms: $\chi_{\left(2\right)}^{2}$ = 0.09; *p* = 0.95] in the final model. Yet, Bayesian analyses moderately supported the data under the null hypothesis for the first (BF_01_ = 4.7), the second (BF_01_ = 5), and the third (BF_01_ = 5.4) time window.

Finally, also for C4 LRT values were non-significant for the first [$\chi_{\left(2\right)}^{2}$ = 0.08; *p* = 0.95], the second [$\chi_{\left(2\right)}^{2}$ = 0.3; *p* = 0.86] and the third [$\chi_{\left(2\right)}^{2}$ = 0.22; *p* = 0.89] time window. Even in this case, Bayesian analyses moderately supported the null hypothesis, indicating no effect of sham tDCS on LMFP computed in this cluster (0–50 ms: BF_01_ = 5.4; 50–100 ms: BF_01_ = 5.4; 100–150 ms: BF_01_ = 5.1).

### Sham stimulation—source modeling analyses

Source modeling analyses on sham recordings mirrored results from the sensor analyses. A full report of statistical results of analyses run on Global and Local SCD is reported in Table 2.

**Table 2:** *p*-values deriving from the sham data extracted from the same analyses described in Table 1.

	**BA 7 L**	**BA 7 R**	**BA 6 L**	**BA 6 R**
**SHAM**				
0–50 (ms)	*p =* 0.174	*p =* 0.081	*p =* 0.117	*p =* 0.237
50–100 (ms)	*p =* 0.109	*p =* 0.319	*p =* 0.149	*p =* 0.088
100–150 (ms)	*p =* 0.966	*p =* 0.992	*p =* 0.998	*p =* 0.995

Global SCD final model did not include *Condition* in any time window, indicating no effect of sham stimulation on cortical excitability (see Figure 4B).

The same holds for both left and right BA7 and BA6, where LRT indicated no inclusion of the factor *Condition* in the final model.

## Discussion

In this study, we ought to investigate the effects on cortical excitability induced by 10 min of cathodal stimulation over the right PPC. To this purpose, we measured TEPs by means of TMS-EEG recordings before, during and 10 min after the end of the stimulation. TMS was applied over the left PPC. As a control condition, 15 participants underwent an additional session in which sham tDCS was delivered. Since not all the participants took part in both this and Romero Lauro et al.'s experiment, we separately compared stimulation effects in the three groups, i.e., anodal, cathodal, and sham. Indices of local and global activity were computed both at the sensors and cortical sources level.

At the sensors level, no significant modulation of cortical excitability was observed during and after cathodal stimulation in comparison to the pre-tDCS session, neither at a global (GMFP) nor at a local level (LMFP for 4 clusters of electrodes). Furthermore, no significant results were found for any of the considered TEPs' temporal windows, namely an early (0–50 ms), a middle (50–100 ms), and a late (100–150 ms) one, chosen to assess different TEP's components. Source modeling confirmed the results observed at the sensor level, since SCD did not change during or after stimulation with respect to the pre-tDCS condition, both when computed at a global level or in BAs, matching the clusters of the LMFP analysis. A similar pattern of results without any significant changes in cortical excitability among the three pre-, during- and post-tDCS conditions was found when sham tDCS was delivered. This result confirms the reliability of the TMS-EEG approach and no effect of test-retest of TEP-derived indices of brain activation (Lioumis et al., 2009; Casarotto et al., 2010; Kerwin et al., 2017).

The absence of significant changes among pre-, during- and after-tDCS conditions by itself does not provide evidence that the three conditions are the same. To further check this chance, a Bayesian analysis was performed to directly test the null hypothesis of no change among the three conditions. Despite subtle differences, all the results of the Bayesian analysis converge in suggesting a moderate indication toward the null hypothesis.

Although results suggest a null effect of cathodal tDCS on cortical excitability, further corroborating evidence from different approaches is needed to support such a negative conclusion. Importantly, evidence of the modulatory effects of cathodal tDCS has been provided so far by different approaches. First of all, a reduction of neurons' firing rate was observed after cathodal tDCS in animal studies (Creutzfeldt et al., 1962; Bindman et al., 1964; Purpura and McMurtry, 1965). Furthermore, when applied over M1, cathodal tDCS resulted in a decrease of corticospinal excitability assessed by MEPs (Nitsche and Paulus, 2000; Lang et al., 2004), led to a widespread decrease of regional blood flow as measured by PET (Lang et al., 2005, but see also Baudewig et al., 2001), and increased the inter-hemispheric coherence of the resting fMRI signal between the left and right homologs regions of the motor system (i.e., M1 and SMA) as well as the functional connectivity in the motor and default mode networks (Amadi et al., 2014).

The conclusion that instead can be firmly drawn from the present set of results is that cathodal effects on cortical excitability differ from the ones induced by anodal tDCS. The present study indeed mirrored a previous one from our group (Romero Lauro et al., 2014, 2015), where the same procedure and data analysis were performed to track the effects of anodal tDCS. These previous results unveiled a significant rise of cortical excitability during and after anodal tDCS at a global level, both when measured by GMPF and by SCD. Analyses at the local level showed that the rise of cortical excitability spread along a bilateral fronto-parietal network, presumably following structural/functional connections along the default mode brain network. The significant findings in these previous studies confirm the feasibility of TMS-EEG approach to tap tDCS effects on cortical excitability. Moreover, TEPs have been shown to be a reliable measure of cortical excitability when the same parameters are maintained (Casarotto et al., 2010). The lack of significant effects in the present study cannot be attributed thus to the experimental paradigm, rather indicates a crucial imbalance between the anodal and the cathodal impact on cortical excitability.

In literature, several studies show an asymmetry between anodal and cathodal tDCS effects, for example in a qualitative review and meta-analysis, Jacobson et al. (2012) revealed how the coupling of anodal-excitatory and cathodal-inhibitory effect is robust in the motor and perceptual domains, but controversial when cognitive functions are addressed. In most of the cases, indeed, when memory, language or, generally, when higher-order cortical regions are tested, as in our study, an excitatory/enhancing effect of anodal tDCS is observed, whereas cathodal tDCS effects are less effective or ineffective. For instance, in a double-blind sham-controlled, within-subjects study, 20 min of anodal but not cathodal tDCS over the left peri-sylvian area improved the performance of healthy subjects in an associative verbal learning task (Flöel et al., 2008). Similarly, the anodal, but not the cathodal, tDCS over the left dorsolateral prefrontal cortex (DLPFC) enhanced the performance at a complex task, such as the remote associates task, involving both language processing and executive abilities (Cerruti and Schlaug, 2009). In the domain of working memory, anodal tDCS over left DLPFC increased accuracy in a sequential letter task whereas cathodal effect did not differ from that of sham stimulation (Fregni et al., 2005). The frequent lack of significant behavioral effects induced by cathodal tDCS is possibly the reasons why many studies, performed both in healthy and clinical population, focused only on testing the anodal vs. sham modality of stimulation (Jacobson et al., 2012). It has to be noted that previous studies investigating tDCS-induced cognitive modulations did not target M1. It follows that cathodal effects highlighted by targeting M1 could be due to a greater sensitivity of this area to cathodal tDCS, having a different cortical organization compared to the rest of the homotypic isocortex.

Nevertheless, there are examples of tDCS effects limited to anodal polarity also in the heterotypic isocortex, such as in the motor (Baudewig et al., 2001; Priori, 2003), visual (Antal et al., 2004; Sczesny-Kaiser et al., 2016), and somatosensory systems (Matsunaga et al., 2004). Within the motor domain, for instance, there is evidence of greater effectiveness of anodal than cathodal stimulation. Indeed, anodal tDCS applied to M1 during task execution enhanced motor speed and dexterity (Nitsche et al., 2003), and also motor learning and adaptation (Nitsche et al., 2003; Boggio et al., 2006; Galea and Celnik, 2009; Hunter et al., 2009; Reis et al., 2009). In contrast, cathodal tDCS showed no effect on learning (Nitsche et al., 2003; Galea and Celnik, 2009; Reis et al., 2009), or on reaction times (Nitsche et al., 2003).

Critically, with a methodology more similar to that applied in the present research, a previous TMS-EEG study (Pellicciari et al., 2013) found the coupling of anodal-excitatory and cathodal- inhibitory modulation on TEPs by stimulating M1; however, also in this case, the effect of the two polarities was different, since increased excitability after anodal stimulation was found over both hemispheres whereas the cathodal stimulation induced opposite effects over the two hemispheres, namely reduced excitability over the stimulated hemisphere and facilitation in the contralateral one.

The mechanisms underlying this asymmetry in the effectiveness of tDCS polarity-dependent effects are still unclear. Jacobson et al. (2012) suggested that the lack of cathodal-inhibitory effects when tapping cognitive functions, as compared to the case of the motor system, could be due to several factors, including the recruitment of broader cortical networks, the greater susceptibility to external noise of the behavioral measured adopted, the influence of the initial activation state and the greater occurrence of bilateral interactions supporting contralateral compensation. Among these explanations, the most compelling seems to be the initial activation state of the target area. More specifically, whereas addressing motor functions could be done using simple tasks (i.e., reaction times) or passively (i.e., recording MEPs), exploring tDCS effects on cognitive functions entails the use of complex tasks, prompting high activations of the target regions. Hence, the anodal-excitatory effect is additive, enhancing the actual activation state level. In contrast, the cathodal inhibitory effect might be counterbalanced by the task-induced activation, leading to null effects. However, the hypothesis of a link among anodal tDCS—increased neuronal excitability/enhanced behavioral performance, at odd with cathodal tDCS—decreased neuronal excitability/reduced behavioral performance, run the risk to oversimplify the pattern of possible behavioral and neurophysiological outcomes, especially outside the sensorimotor domain. This simplistic proposal does not take into account the complex combination of excitatory and inhibitory connections within broader cortical networks, the dependence upon the network state activation, the level of performance or task engagement, the well-known inter-individual variability and other factors affecting the current diffusion in the brain, such as different neural population and orientation (see Fertonani and Miniussi, 2017).

An alternative explanation is that the effects of tDCS may depend in an opposite way on the background level of activity in the system (Matsunaga et al., 2004). For instance, if cathodal stimulation reduces the level of neural discharge, then it may produce effects in systems with high levels of basal activity. However, if the resting state is characterized by low levels of spontaneous discharge, then cathodal tDCS may have little or no effect.

For these reasons, a possible explanation about the absence of effects induced by cathodal tDCS in our study could be found in the computational model describe in Lafon et al. (2016).

In this study, a combination of computational modeling and *in vitro* experiments were used to explore how Direct Current Stimulation (DCS) affected the neuron's input/output function, namely the synaptic efficacy (input, I) and the likelihood of eliciting an action potential (output, O), by modeling a 2-compartment neuron, including the soma and the dendrites, and by recording *in vitro* from hippocampal pyramidal cells.

Results showed that opposing polarization of soma and dendrite may account for the asymmetry in the strength of the effects of stimulation for opposite polarities. In particular, anodal tDCS modulates I/O functions by increasing the likelihood that neurons elicit an action potential in response to a fixed input and, in addition, increasing the synaptic current entering the cell. The opposite occurs for cathodal stimulation, i.e., a decrease in output likelihood by soma hyperpolarization. However, in cathodal tDCS this effect is canceled by the depolarization of the dendrites, hence increasing the probability of spike initiation at this location. Therefore, cathodal, compared to anodal, tDCS creates an opposite shift in the threshold of I/O function, but with a weaker impact because of these two counterbalancing opposite effects on the two neuron's compartments.

By applying this model to the conventional M1-SO electrodes' montage, Lafon et al. (2016) analyzed which portion of cortical tissue in human cortex is exposed to tangential or radial current flows. In particular, they simulated and averaged the population effects on the I/O function for the anodal or cathodal tDCS. Data suggest that while anodal stimulation induces an increase in the population-level I/O function, cathodal tDCS do not modulate neuronal efficiency at the population level.

A polarity asymmetry has also been shown in the induction of LTP effects. For instance, in M1 mice slices, only anodal, but not cathodal, DCS coupled with simultaneous synaptic activation was able to induce LTP (Fritsch et al., 2010). In a similar vein, in a recent study (Kronberg et al., 2017) DCS applied during plasticity induction on rat hippocampal slices resulted in asymmetric effect on synaptic plasticity since both anodal and cathodal enhanced LTP and reduced LTD. Crucially and in line with Lafon's et al. results, this study unveiled how the effect of DCS are dependent upon the location and frequency of active synapses, more than from the polarity of stimulation.

Another important point is that in our study TMS-EEG recordings were collected while our participants were in a resting state. We have recently shown how cortical excitability, as measured by means of TEPs, is modulated by the activation state of the target area (Pisoni et al., 2017). In particular, during task execution the induced rise of cortical excitability spread following functional rather than structural connections, encompassing only task-relevant brain regions. Whether cathodal effects on cortical excitability would be different by administering a task involving the target region is a crucial question, calling for further research.

Possible limitations could derive from the dimensions of the electrodes and the montage used here. In a recent study, indeed, Roy et al. (2014) used a combination of EEG and high-definition tDCS, with a 4 × 1 ring electrodes configuration. For future studies, this could be an ideal montage to better observe the real-time effects of tDCS on cortical excitability. In line with this observation, another indication for future studies could be to use a high-resolution tDCS to probe with TMS the same tDCS target area. Finally, as highlighted by Saturnino et al. (2015), the main tDCS effects might be between the two electrodes, thus directly stimulating with TMS the region targeted with the cathode could lad to different results. Future research is then needed to explore this issue, noting that in the case of anodal stimulation even when targeting the contralateral homologous brain region TMS-EEG highlighted a vast change in cortical excitability.

In conclusion, the results of the present study show no significant modulation of cortical excitability as measured by TEPs, when the cathodal tDCS is applied over the right parietal cortex. In contrast, previous studies showed a significant rise of cortical excitability both stimulating the same area at resting state (Romero Lauro et al., 2014, 2015), or a different one during task execution (Pisoni et al., 2017). Taken together these results show an asymmetric impact of anodal and cathodal stimulation on cortical excitability, in line with previous behavioral, neurophysiological and computational modeling studies. This asymmetry warrants further research to better understand the underlying mechanism and should be taken into account in study design for both research and clinical purposes.

## Author contributions

EV, AP, GM, AV, and LR: designed the study; EV, AP, GM, AV, AG, and LM: performed the experiments; EV, AP, and LR: analyzed the data; EV, AP, and LR: mainly contributed to the manuscript; GM, AV, MR, NB, and GV: contributed to the manuscript.

### Conflict of interest statement

The authors declare that the research was conducted in the absence of any commercial or financial relationships that could be construed as a potential conflict of interest.
